# Institutional Outdoor Work and Administration

**Published:** 1911-09-23

**Authors:** 


### SPECIAL INSTITUTIONAL ARTICLE.

### INSTITUTIONAL OUTDOOR WORK AND ADMINISTRATION.

By A SANATORIUM SUPERINTENDENT.

**[The Editor will be pleased to receive criticisms and suggestions of the application of the principle underlying this article. So many institutions have their own methods for producing various needs that an interchange of experience would be of the greatest value to the whole institutional world.]**

### III.—POULTRY.

At many institutions the poultry yard is a most miserable-looking sort of place. Both house and run are too small for the numbers kept, the grass has long ago been all scratched away, and the fowls are curiously mongrelised. Being unsuitably fed to boot, they pine and mope, and both for purposes of egg-production and for the table give about as unsatisfactory a return as possible. On the other hand, it must be admitted that life itself, let alone an institutional matron's spare time, is too short for the fussing attention that a real fancier goes in for. To strike the useful and practicable mean between these two extremes is obviously the aim to be fixed, and to achieve it one must first take into account local conditions, which, as will be seen, affect procedure considerably.

#### Scope of Operations.

To begin with, how much does one think of doing? Let us take yet again the case of a first-class fifty-bed sanatorium, using sixty eggs a day and able to consume "chicken diet" up to the full extent, which, after some difficult enough item-dissection, is found to effect a saving on the butcher's bill. Now the first of these requirements alone entails a rather elaborate plan of campaign. Sixty eggs a day is, roughly, twenty-two thousand a year. Excluding prize-winning strains, it is not safe to expect hens to average more than 120 eggs per annum each. Therefore 183 laying hens* are needed for a start. And this 120-egg yield will only be given in the hen's first two seasons, after which it is customary to kill her. So that every two years the stock has to be replenished, and 183 table fowls, which will make, say, four "diets" each, can be written off against the cost of renewal. Such cost, buying cross-bred pullets at 3s. per head, will come to £27 9s.—rather a large sum to ask of one's probably technically uninformed but certainly frugally-minded committee, every other year, and for stock alone. It will be better to keep a note and a hold in the memory of the above calculation, but to begin cautiously in a smaller way, a way which may yet end in something bigger than ever.

#### Other Local Conditions.

Next, can the birds be given their liberty, or must they be kept penned up? The answer will largely decide the choice of breed (or first cross) to be kept, since most heavy varieties in captivity soon get too fat to lay well. A good food supply of table scraps (fairly divided with the pigs) and some cabbage leaves from the garden we are taking as granted; also help for the matron from female patients, and from a man employed on the place in the case of rough or dirty work. It is further settled, too, by the nature of the case that we are what is known as "utility" people, and do not aspire to exhibit at shows; further, that the main purpose is egg production. Then the locality—is it high and bare, or warm and sheltered? in the North or in the South? If the former, we must have hardy kinds in order to do well—Redcaps, Anconas, Rhode Island Reds, Plymouth Rocks.

#### A Modest Beginning.

The first thing, then, having reckoned up unalterable conditions, is to get to know a little of the subject by expending a penny on the current number of the *Feathered World*, and selecting a handbook from those found mentioned. There is a great deal to learn, and subsequently a great deal to do. A supply of eggs all the year round means keeping different kinds. One's Croad Langshan or Partridge Wyandotte breed or cross will lay well in winter, while the familiar Leghorns come in in the summer time. In the spring the egg question settles itself. Perhaps the best stock to start with is fifty cross-bred pullets and a pen of some pure variety, obtained from a firm of standing. In the matter of housing and feeding these there are four things to beware of—namely, overcrowding, overfeeding, no grit, and not enough water. First, as regards housing. Buy from a leading maker a combined roosting house and scratching shed accommodating twenty birds, the maximum number which should be together under one roof. This can be duplicated perhaps, by a handy man, out of odd material to be found about an institution, such as old flooring boards, surplus light planking, piano crates, etc. "Slabs," the bark-covered selvedge of logs of timber, can be got very cheaply from a sawmill and may be worked in with a little ingenuity, interstices being stopped up inside to prevent draughts, and a good coat of whitewash rendering the interior uninviting to vermin. The novice will judge scratching sheds an extravagance, but, as a matter of fact, they are essential to winter egg production, which is most important of all; their principle is, of course, to give fowls sheltered exercise on stormy days. Have dropping boards under

* In this connection the recent report of one of our sanatoriums is a little amusing. Its "small poultry farm" yielded 2,500 eggs in the twelve months. Division of this total by 120 will show that exiguity may certainly, as logicians say, be predicated here.

the perches and chaff on the floor of the house. Clean the former weekly, the latter every two months or so. Fowl manure is of the richest, and needs mixing with earth before use.

### Feeding and Management are best studied in manuals, but as bearing on our present case it may be said that there is no better morning feed than the scraps. Remember that a fat hen will not lay, and that most hens at liberty with access to grass are feeding themselves all day long. A bone cutter is almost an essential, green bone being used, as cooking extracts all the virtue. Grit crushers are sold, but the best instrument is the garden roller, which will convert the broken crockery the domestics are sure to supply constantly into an excellent digestive adjuvant. Fresh water must be given often and kept shaded from the sun. A stream running through a wood (an excellent place for fowls) is the ideal arrangement. Experience will soon make the keen learner competent in the elements of the subject, and then probably, since Nature has implanted in the female disposition a taste for rearing young things, efforts will be made to hatch some chicks under "clockers" or by use of an incubator. Now no one need fear not being able to make a good incubator answer. Artificial brooders are indeed difficult at first, but if the broad rule be adhered to of hatching with incubators and giving the chicks (after at least thirty-six hours in the drying chamber) to broody hens to rear, very good results can be got—at the price, certainly, of a vast deal of trouble. But, as we have seen, there is somewhat of a natural provision that this trouble will be easily borne, and it becomes much lessened by experience and routine.

### What to Work up to (in a Sanatorium).

Probably not altogether pleasant experience of buying eggs for setting will soon induce the wish for enough breeding hens to supply eggs for the incubators, in addition to those for current consumption. Every spring, of course, one must hatch chickens, because, as we have seen, every second year the stock of laying hens has to be renewed. To have eggs ready to set is the greatest of advantages, as one can be sure that the male bird is unrelated to the hens, that all are mature, that they have either full liberty or a large grass run, and that the eggs are not shaken and are set when not more than thirty-six hours old. Given these conditions, the result is a 90 per cent. hatch of large, vigorous chicks, that thrive wonderfully given half a chance. But clearly a numerous stock is needed for this. The aim with which to stimulate a matron's ambition is to have, say, two hundred-egg incubators going by the end of January, to hatch out rapidly what she needs for maintenance of laying and of breeding stock, separating the cross-bred cockerels for fattening and home consumption, and selling the surplus pure-bred ones by advertisement. In May it is time to stop: late hatched chickens are not profitable, for reasons explained in the elementary literature of the subject. Thenceforward till, say, September she should hatch Aylesbury duck eggs. A ten-weeks Aylesbury duckling will make six "diets," while a three months' chicken, on the other hand, on an average hardly makes three.

But before now, of course, a whole-time poultry woman will have been found necessary; and here is ideal work for a convalescent consumptive, of which class, indeed, there are already a good number in the poultry business. A matron's time will have been well spent if she can thus provide employment for patients; and the work is in close relation to the housekeeping, which in a sanatorium, even more than in other medical institutions, is bound to absorb much of her attention. As regards figures, it is impossible to make a general case serve all the varying local conditions. But given persevering management, then no rent to pay, a market on the spot, and some free food supply and labour, are advantages making it certain that institutional poultry will pay well. Not so well as pigs, because fowls are many times the amount of trouble; but still, they pay well, and with the steady rise in the price of eggs should tend to pay still better.

